# Evaluating Response of Mass-Reared and Irradiated Navel Orangeworm, *Amyelois transitella* (Lepidoptera: Pyralidae), to Crude Female Pheromone Extract

**DOI:** 10.3390/insects11100703

**Published:** 2020-10-15

**Authors:** Joshua Reger, Jacob Wenger, Gurreet Brar, Charles Burks, Houston Wilson

**Affiliations:** 1 Department of Plant Science, California State University, 2415 E San Ramon, Fresno, CA 93740, USA; jawenger@csufresno.edu (J.W.); gurreetbrar@mail.fresnostate.edu (G.B.); 2 Department of Entomology, University of California, 900 University Ave, Riverside, CA 92521, USA; houston.wilson@ucr.edu; 3 USDA, Agricultural Research Service, San Joaquin Valley Agricultural Sciences Center 9611 South Riverbend Avenue, Parlier, CA 93648-9757, USA; charles.burks@usda.gov

**Keywords:** sterile insect technique, wind tunnel, *Amyelois transitella*, almonds, pistachios, Lepidoptera, Pyralidae

## Abstract

**Simple Summary:**

The navel orangeworm is an important pest of almonds and pistachios in California. Sterile insect technique (SIT) is being explored as an additional component of management of this pest. Preliminary field releases of sterile navel orangeworm shipped from a facility in Phoenix, AZ resulted in poor recovery of males in pheromone traps, raising concerns about the mass-reared male moths’ quality. In this study, a wind tunnel was used to evaluate the response of irradiated and non-irradiated mass-reared navel orangeworm males to pheromone extract from females, and their performance was compared to two strains of locally reared non-irradiated navel orangeworm. Initial responses were similar for all moths tested. A lower proportion of mass-reared moths contacted the pheromone source. The underlying mechanism for this reduction remains unclear, but is likely related to damage incurred during the mass-rearing and shipping process. Our findings indicate navel orangeworm in the current program is generally competent to locate a sex pheromone source, but the rearing and transportation protocols may need refining.

**Abstract:**

The navel orangeworm, *Amyleois transitella* (Lepidoptera: Pyralidae), is a key pest of almonds and pistachios in California. Larvae directly feed on nuts, reducing quality and yield, and adults can introduce fungi that produce aflatoxins. The development of sterile insect technique (SIT) is currently being explored as a management tool for this pest. Large quantities of *A. transitella* are mass-reared, irradiated, and shipped to California from a USDA APHIS facility in Phoenix, AZ. Preliminary field releases of sterile *A. transitella* from this facility resulted in poor recovery of males in pheromone traps, raising concerns that mass-reared male *A. transitella* may not be responding to pheromone from virgin females. In this study, a wind tunnel was used to evaluate the response of both irradiated and non-irradiated mass-reared *A. transitella* males to crude pheromone extract from females, and their performance was compared to two strains of locally reared non-irradiated *A. transitella*. While initial responses associated with pheromone detection where similar between mass-reared and locally reared moths, a lower proportion of the mass-reared moths ultimately made contact with the pheromone source. Surprisingly, the addition of irradiation did not further decrease their performance. While mass-reared moths respond to pheromone, their ability to locate and make contact with the pheromone source appears to be impeded. The underlying mechanism remains unclear, but is likely related to damage incurred during the mass-rearing and shipping process.

## 1. Introduction

Sterile inset technique (SIT) can be used for the area-wide prevention, eradication, or suppression of insect pests in agricultural, natural, and urban environments [1]. In this approach, sterilized insects are mass-reared and released to mate with wild conspecifics, which can lead to population reductions over time [2]. Lepidoptera have become an increasing target of SIT due to their growing impact on high-value agricultural crops around the world [3,4,5], and SIT has been successfully used as part of an area-wide integrated pest management (IPM) program to control multiple lepidopteran pests such as codling moth *Cydia pomonella* (L.) (Lepidoptera: Tortricidae) [6], painted apple moth *Teia anartoides* (Walker) (Lepidoptera: Erebidae) [7], and pink bollworm *Pectinophora gossypiella* (Saunders) (Lepidoptera: Gelechiidae) [8]. The success of an SIT program is dependent on a variety of factors, but the competitiveness of mass-reared sterile insects is critical, and can be influenced by rearing conditions, sterilization process, transport and release methods. As such, a variety of laboratory assays are necessary to verify moth quality and performance throughout program development, including the use of wind tunnels [9].

The navel orangeworm, *Amyelois transitella* (Walker) (Lepidoptera: Pyralidae), is the key pest of California almonds and pistachios. Adults deposit eggs on the nuts, and the larvae directly feed on the nut meat, which reduces crop yield and quality. Additionally, *A. transitella* adults can introduce the fungus *Aspergillus flavus*, which produces aflatoxins [10,11], known human carcinogens that are highly regulated in key export markets [12]. Currently, California growers have an extremely low threshold for *A. transitella* damage and typically aim for ≤2% infestation. This pest overwinters as larvae in remnant “mummy” nuts left behind in the orchard after harvest. As such, orchard sanitation is the foundation of *A. transitella* management, along with well-timed insecticide applications, mating disruption and timely harvest [13].

Recently, researchers have started to explore the development of SIT for *A. transitella* as well. This effort makes use of a mass-rearing and irradiation facility in Phoenix, AZ that is operated by the United States Department of Agriculture (USDA) Animal and Plant Health Inspection Service (APHIS) Plant Protection and Quarantine (PPQ). This facility was originally designed for production of *P. gossypiella* as part of an area-wide SIT program initiated in the late 1960s [14]. With the successful eradication of *P. gossypiella* in 2018, efforts were initiated to explore the use of this mass-rearing facility for production and sterilization of *A. transitella*.

Initial pilot studies in 2018 evaluated recapture of mass-reared and irradiated *A. transitella* in small (2 acre) and large (640 acre) pistachio orchard plots. In the small plot, moths were released on the ground by hand using metal trays suspended from the tree canopy. In the large plot, moths were released aerially from a modified small airplane. Multiple releases were made at both sites, but all of them resulted in very low recapture (<0.001%) of male moths in wing traps (Pherocon 1C, Trece Inc., Adair, OK, USA) baited with pheromone lures (Biolure, Suterra, Bend, OR, USA) [15]. Such poor recapture of the sterile *A. transitella* could be due to a variety of factors associated with the mass-rearing, sterilization, transportation and release process. More specifically, the absence of a flight component from the mass-rearing process poses the risk of selecting against moths that can adequately detect and follow pheromone plumes. Here, in order to verify that mass-reared male *A. transitella* would indeed respond to female *A. transitella* pheromone, a wind tunnel assay was used to evaluate the effects of moth strain, rearing conditions and irradiation, on male response to crude pheromone extract from females.

## 2. Materials and Methods

### 2.1. Insect Cultures

In total, there were four moth types flown in this assay that included (1) locally-reared non-irradiated Mendota strain moths (“Mendota-Local”, *n* = 103), (2) locally-reared non-irradiated Phoenix strain moths (“Phoenix-Local”, *n* = 89), (3) mass-reared non-irradiated Phoenix strain moths (“Phoenix-Facility”, *n* = 101) and (4) mass-reared irradiated Phoenix strain moths (“Phoenix-Facility-Irradiated”, *n* = 88).

Locally reared moths were sourced from *A. transitella* colonies maintained at the USDA Agricultural Research Service (ARS) San Joaquin Valley Agricultural Science Center (Parlier, CA). The “Mendota” strain was established in 2010 from *A. transitella* eggs collected from an almond orchard in Fresno County, and was refreshed with additional eggs from the same site in 2011. The “Phoenix” strain was established in 2018 using *A. transitella* larvae from the USDA-APHIS mass-rearing facility in Phoenix, AZ. Locally reared *A. transitella* were grown in 1-gallon glass jars on an artificial wheat bran diet [16] at 26 °C in a 14:10 (L:D) photoperiod. Late instar male larvae were sexed twice a week and isolated until adults emerged in a reverse photoperiod. The reverse photoperiod had an inverse L:D cycle with scotophase from 2 a.m. to 12 p.m., which allowed moths to be flown in the last 3 h of scotophase beginning at 9 a.m.

Irradiated and non-irradiated mass-reared adult *A. transitella* were sourced from the USDA APHIS mass-rearing facility, where they were also reared on a wheat-bran diet. *Amyelois transitella* at the APHIS facility emerge in darkness with a single light source to lure them into a vacuum tube collection system. The tubes transport moths to a cyclone chamber, where there are exposed to cold air (5 °C) to immobilize them. Cohorts of roughly 1000 moths (<24 h old, 50:50 M:F) were placed into a large Petri dish and prepared for shipment. Moths in the irradiated treatment were additionally exposed to 300 Gy of gamma radiation from a Cobalt-60 core. APHIS facility moths were then shipped overnight in a cooler held close to 5 °C. Upon arrival at the USDA-ARS San Joaquin Valley Agricultural Science Center, each Petri dish was emptied into a 30 × 30 × 30 cm cage (BugDorm-1, MegaView Science, Taiwan). After being transferred to the cage, moths were provided 10–30 min to move from the Petri dish to the walls of the cage. Fifteen virgin males from each treatment were then selected from individuals perched on the side of the cage. The male moths were placed in a growth chamber with the local strains and allowed 48 h to adjust to the reverse photoperiod.

### 2.2. Gland Extraction

Male moths were flown in a wind tunnel to a female *A*. *transitella* crude pheromone gland extract. Crude pheromone extract is a complete attractant known to produce highly replicable results with *A. transitella,* which is why it was selected for this study [17,18,19]. The gland extract was prepared using 1–5 day old adult calling females from the “Mendota” colony [17,18,19]. Pheromone glands were prepared in 150 µL of hexane containing 60 pheromone glands. The concentration of the main pheromone component (Z11, Z13–16: Ald) was verified via gas chromatography for each 150 µL batch. Verification was conducted with a Hewlett Packard model 6890 gas chromograph. Concentration of Z11, Z13–16: Ald was determined from a 10 µL aliquot subjected to the following temperature program: injection temperature of 80 °C, a temperature increase of 50 °C/min to 210 °C with a 7 min hold, then a temperature increase of 50 °C/min to 240 °C with a 5 min hold with helium as the carrier gas. Any batch that did not meet the minimum concentration of 0.5 µg/L of Z11, Z13–16: Ald was supplemented with additional glands. All pheromone solutions were stored in the freezer when not in use.

### 2.3. Wind Tunnel

The wind tunnel was housed in an isolated environmentally controlled room with no exterior windows and black vinyl strip door. The tunnel consisted of a Plexiglass rectangular enclosure measuring 98 × 98 × 250 cm with 5.08 cm circular red discs on the floor (25 discs per m^2^) [20]. During flights, the air speed was 75 cm s^−1^ and room held at 24–26 °C, >50% RH, and illuminated at 1–2 lux.

### 2.4. Assay Setup

Moths were individually flown in the wind tunnel during the last 3 h of scotophase, mimicking the time females naturally call for males in the orchard [21]. The afternoon before a flight date, 5–10 virgin males of each of the four moth type treatment groups were transferred from their holding cages and individually isolated in 2 × 2 × 4 inch wire mesh cages (i.e., 20–40 individually caged moths total that were flown individually on a single date). On the morning of a flight session, the wind tunnel room was set to optimal environmental conditions. Once conditions were obtained, the tray of individually caged moths was moved into the wind tunnel and allowed to adjust to the environment for 1 h.

A pheromone lure was prepared by pipetting 25 µL of pheromone extract or 12 ng of Z11, Z13–16: Ald on a hexane soaked 1 cm circle of #1 Whatman filter paper. The lure was mounted vertically to a 25 cm tall wire stand placed 169 cm upwind in the tunnel. Higher concentrations of solvent are released from the lure in the first few minutes. Consequently, the lure was allowed a 5 min off-gassing period after placement in the tunnel [18]. Each lure was used only once and for no longer than 35 min, which was the longest run time for a single session.

On each date, at least five males of each of the four moth-type treatment groups were individually flown. The order of moth type was randomized on each date. All moths from each treatment group (5–10 moths) were flown individually before a new moth type was tested so that behaviors could be clearly observed. During each flight session, five behaviors were measured for each moth; this included no response, wing fanning, taking flight, locking onto plume, and contact with the lure. Locking onto plume was defined when moths began to make a straight flight path towards and level with the lure. Each individual moth was given 1 min to initiate any behavior and transition to the next subsequent behavior. If no behavior or no new behavior was observed, the moth was removed and the absence of behavior recorded. No behavior after contact with the lure was recorded, although male *A. transitella* are known to perform additional mating rituals once contact with a female has been made [22].

### 2.5. Data Analysis

Mate-seeking behaviors were analyzed using the R Statistical Program (3.5.1, http://www.r-project.org/). Data were evaluated with generalized linear mixed models using the “lme4” package with a binomial distribution and logit link. Each of the five behavioral responses were analyzed separately. For each analysis, the proportion of total moths that exhibited the specific behavioral response were evaluated relative to fixed effects “Moth Strain”, “Rearing Method”, and “Irradiation”. Models included the random effect “Moth Type” nested within “Date”, since fixed effects were not fully crossed across the four moth types. In each model, significance (*p* ≤ 0.05) of fixed effects was determined by Pearson’s χ^2^ test using the “drop1” function.

## 3. Results

**Figure 1:**
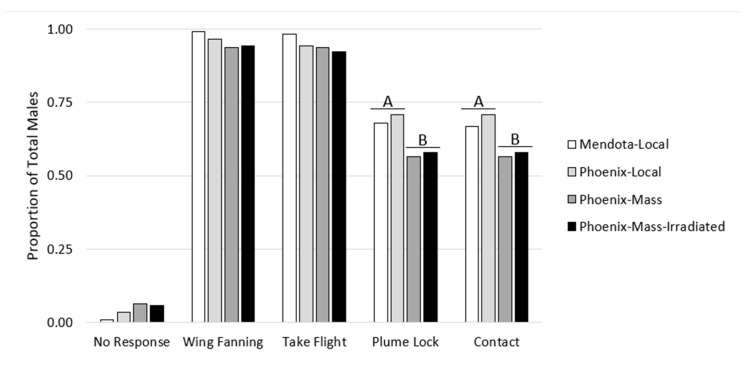
Proportion of male *A. transitella* exhibiting each mate-seeking behavior. Letters indicate significant differences between local and mass-reared moth types.

A total of 372 moths were flown over 12 separate dates. While generally a majority of individuals displayed the preliminary mate-seeking behaviors of wing fanning and taking flight (92–99%), fewer moths managed to successfully lock onto plume and contact the lure (56–71%). Only 1–6% of moths exhibited no behavior at all. Moth strain and irradiation had no influence on any of the measured behaviors, whereas mass rearing appeared to reduce the proportion of moths that successfully locked onto the plume and made contact with the pheromone source (Table 1, Figure 1).

## 4. Discussion

The success of a SIT program is dependent on sterile males locating and mating with wild female conspecifics, and the ability to do so can be affected by multiple aspects of the rearing, transport and release process [23]. For Lepidoptera in particular, it is critical that males are able to effectively detect, respond to and follow pheromone plume trails [24,25]. Here, findings indicate that the *A. transitella* production and transportation methods currently utilized appear to negatively influence the ability of males to adequately locate females.

Wing-fanning is an initial response of male *A. transitella* indicating that the female sex pheromone has been detected [21,26]. In other species such as *P. gossypiella* and *Grapholita molesta* (Lepidoptera: Torticidae), this behavior is correlated with a higher rate of success contacting a pheromone source [27,28]. While this was the case for locally reared *A. transitella*, mass-reared moths from the USDA APHIS facility exhibited wing fanning and took flight but were less successful at locking onto the plume and making contact with the pheromone source. As such, the mass-reared and shipped moths appear to detect pheromone but seem to have a reduced ability to follow plumes and make source contact.

Without adequate maintenance of genetic diversity or built-in processes to preserve key performance features, mass-rearing can potentially place selection pressure on organisms towards traits associated with the rearing environment itself, which can in turn detract from field performance. In the current mass-rearing process, dozens of virgin male and female *A. transitella* are mated in 1-gallon glass jars, and females subsequently deposit eggs onto paper material that covers the top of the jar [29]. In such an enclosed environment, there is little need for males to follow pheromone plumes over long distances, and in this may present a selective force against long-distance plume-following behavior. Alternately, mass-reared females may evolve to express an abnormal blend of pheromone that in turn alters male response to synthetic and/or wild moth pheromone, which has been observed in other Lepidoptera [27,30,31]. Here, both locally reared “Mendota” and “Phoenix” strains of *A. transitella* responded to pheromone, while mass-reared moths were less successful in actually locating and making contact with the source, suggesting that the “Phoenix” strain pheromone response remains intact but mass-rearing of this strain seems to have a negative impact on their ability to fly towards and make contact with the pheromone source. That said, both “Mendota” and “Phoenix” are laboratory strains, and a comparison of their performance with wild-collected *A. transitella* males would be a more accurate evaluation. Another alternate approach would be to compare the attraction of wild male *A. transitella* to pheromone extract from “Phoenix” strain versus wild-collected females.

The mass-rearing and shipping process can also result in physical damage to moths associated with collection, handling, and transportation to release sites. While both local and mass-reared *A. transitella* were reared at constant optimal temperatures, upon emergence in the USDA APHIS facility the mass-reared adult *A. transitella* was aggregated through a vacuum collection system and then rapidly chilled and held at 2–10 °C for over 24 h. Susceptibility of adult *A. transitella* to chill injury remains unclear, but in other lepidopteran programs this has been shown to be an important factor that can influence moth performance [32,33,34]. The effect of cold temperatures on *A. transitella* has thus-far only been explored as a post-harvest management strategy, with data indicating that eggs and larvae are more susceptible to mortality from cold temperatures than pupae or adults [16,35]. In addition to cold chilling, the mass-reared moths were also subjected to a long-distance shipping process, which can have variable impacts due to vibrations and changes in atmospheric conditions. For instance, false codling moth, *Thaumatotibia leucotreta* (Lepidoptera: Torticidae) flight ability and longevity were all negatively impacted by long-distance transport [36], whereas in contrast shipping pupae and adults of *C. pomonella* from Canada to South Africa did not seem to have any effect on emergence, longevity or ability to mate [37].

Surprisingly, the addition of irradiation did not appear to further deteriorate moth performance. The first study of *A. transitella* radiation biology found that full sterility was induced when adults were gamma-irradiated at 540 Gy using a Cobalt-60 source [38]. At this dose rate, the total number of matings per male was only slightly lower than non-irradiated controls. In contrast, a more recent study that utilized X-ray irradiation found that mating frequency of males following irradiation was negatively impacted at doses >125 Gy [39]. In this current study, males were gamma-irradiated at 300 Gy using a Cobalt-60 source, and the impacts of irradiation on male response were confounded by the mass-rearing effect. As such, impacts of irradiation remain unclear and merit further evaluation. For instance, it may be that irradiation does have a negative impact on male performance in the wind tunnel, but here the effect was masked by the stronger negative effects associated with mass-rearing and shipping.

As with other laboratory assays, results from this wind tunnel study may overestimate *A. transitella* performance due to the artificial nature of the testing environment. Furthermore, selection of healthy perched mass-reared moths may have actually underestimated the negative effects of the mass-rearing, irradiation and shipping processes, as the strongest moths from these cohorts were selected for study. Even so, the mass-reared moths used in this assay clearly did not perform as well as the locally reared controls. While findings presented here do indicate that the “Phoenix” strain of *A. transitella* do appear to respond to pheromone cues, modifications to the mass-rearing and transportation process will be necessary to further optimize their performance.

## 5. Conclusions

As with other laboratory assays, results from this wind tunnel study may overestimate *A. transitella* performance due to the artificial nature of the testing environment. Furthermore, selection of healthy perched mass-reared moths may have actually underestimated the negative effects of the mass-rearing, irradiation and shipping process, as the strongest moths from these cohorts were selected for study. Even so, the mass-reared moths used in this assay clearly did not perform as well as the locally reared controls. While findings presented here do indicate that the ‘Phoenix’ strain of *A. transitella* do appear to respond to pheromone cues, modifications to the mass-rearing and transportation process will be necessary to further optimize their performance.

## Figures and Tables

**Table 1:** Summary of analysis (χ^2^ values) to determine influence of strain, rearing method, and irradiation on male *A. transitella* response to crude pheromone extract (*n* = 372).

Fixed Effect	Behavioral Response				
	No Response	Wing Fanning	Take Flight	Plume Lock	Contact
Strain	1.22	1.22	1.59	1.19	1.19
Rearing	1.12	1.12	0.36	4.09 *	4.09 *
Irradiation	0.09	0.09	0.00	0.04	0.04

* *p* < 0.05.
